# The Perceived Impact of COVID-19 on Student Well-Being and the Mediating Role of the University Support: Evidence From France, Germany, Russia, and the UK

**DOI:** 10.3389/fpsyg.2021.642689

**Published:** 2021-07-12

**Authors:** Maria S. Plakhotnik, Natalia V. Volkova, Cuiling Jiang, Dorra Yahiaoui, Gary Pheiffer, Kerry McKay, Sonja Newman, Solveig Reißig-Thust

**Affiliations:** ^1^Department of Management, HSE University, Moscow, Russia; ^2^Department of Management, Kedge Business School, Talence, France; ^3^Department of Management, Kedge Business School, Marseille, France; ^4^Hertfordshire Business School, University of Hertfordshire, Hatfield, United Kingdom; ^5^Department Business and Economics, Berlin School of Economics and Law, Berlin, Germany

**Keywords:** COVID-19, university students, subjective well-being, university success, job prospects

## Abstract

The rapid and unplanned change to teaching and learning in the online format brought by COVID-19 has likely impacted many, if not all, aspects of university students' lives worldwide. To contribute to the investigation of this change, this study focuses on the impact of the pandemic on student well-being, which has been found to be as important to student lifelong success as their academic achievement. Student well-being has been linked to their engagement and performance in curricular, co-curricular, and extracurricular activities, intrinsic motivation, satisfaction, meaning making, and mental health. The purpose of this study was to examine how student perceptions of their degree completion and future job prospects during the pandemic impact their well-being and what role university support plays in this relationship. We used the conservation of resources theory to frame our study and to develop five hypotheses that were later tested via structural equation modeling. Data were collected from 2,707 university students in France, Germany, Russia, and UK via an online survey. The results showed that university support provided by instructors and administration plays a mediating role in the relationship between the perceived impact of COVID-19 on degree completion and future job prospects and levels of student well-being. Student well-being is decreased by their concerns for their degree completion but not by their concerns for future job prospects. In turn, concerns for future job prospects affect student well-being over time. These results suggest that in a “new normal,” universities could increase student well-being by making support to student studies a priority, especially for undergraduates. Also, universities should be aware of the students' changing emotional responses to crisis and ensure visibility and accessibility of student support.

## Introduction

Student well-being has become a concern for many colleges and universities globally as they acknowledge the importance of a balance between psychological, social, emotional, and physical aspects of student lives (e.g., Flinchbaugh et al., 2012; Mahatmya et al., 2018). Student well-being could be understood as “reduction in stress, enhanced experienced meaning and engagement in the classroom, and ultimately, heightened satisfaction with life” (Flinchbaugh et al., 2012, p. 191). Student well-being includes concepts of motivation, identity, self-esteem, self-efficacy, and self-regulation in the context of learning and matriculating through the program to get a degree (Willis et al., 2019). Student well-being has shown to increase their engagement in learning activities, meaning making, a sense of belonging, positive relationships with others, autonomy, and competencies (Sortheix and Lönnqvist, 2015; Baik et al., 2016; Cox and Brewster, 2020) and reduce their burn-out, stress, frustration, dissatisfaction, and withdrawal from active learning (Flinchbaugh et al., 2012; Mokgele and Rothman, 2014; Yazici et al., 2016). Therefore, well-being not only fosters student academic achievement, but also prepares students for lifelong success (Mahatmya et al., 2018). Not surprisingly, many universities across the globe have decided to make well-being their central strategic goal. For example, in Europe, seven universities from seven different regions along with over 100 partnering organizations formed the European University of Well-Being—EUniWell—to promote well-being of students, staff, and communities. Meanwhile, Schools for Health in Europe Network Foundation (2019) is working on health and well-being standards and indicators that offer guidelines to promote health in schools in Europe. In the United Kingdom, the Higher Education Policy Institute (2019) and Advance Higher Education work together to monitor student well-being by continuously collecting and analyzing data from full-time undergraduate students. In the United States, George Mason University, VA, has implemented a university-wide “Well-Being University Initiative” that is coordinated and advanced by a specially created center. The University System of Georgia, USA, has adopted a similar vision of a well-being culture to enhance lives of its community.

Prior to the pandemic, levels of well-being among college students were troublesome (Poots and Cassidy, 2020). For example, in the United States, only one in 10 students graduating from universities measured high in all elements of well-being (Gallup, 2020). In the United Kingdom, undergraduates were reported to have lower well-being than the general population and their well-being was in decline for several years (Higher Education Policy Institute, 2019). This unfortunate state of well-being among students undoubtedly has been devastated by the pandemic that has brought suffering, frustration, discomfort, fear, loss, and other negative emotions and experiences. Students across the world have suddenly been expected to work and learn online, which requires access to good IT infrastructure and equipment, connectivity, and different digital and cognitive skills. Students worry not only about the infection risk but also about their degree completion and unemployment upon graduation, which impacted their well-being even prior to the pandemic (Moate et al., 2019).

Since the outbreak of Covid-19, research has shown the psychological impact of the pandemic on university students and discussed the coping solutions. For instance, disruptions in academic processes due to Covid-19 pandemic have increased student anxiety (Wang et al., 2020), especially for those without adequate social support (Cao et al., 2020). Other health risks, such as depression, alcohol and drug consumption, and eating disorder symptoms, have been reported among German university students (Kohls et al., 2020). Consequently, students with lower levels of mental well-being experience more stress about their academic activities and decreased self-efficacy, satisfaction with coursework, and sense of belonging to university (Capone et al., 2020). Stress also has been found to decrease medical students' enthusiasm to learn and practice medicine upon graduation (Ye et al., 2020). The pandemic has also increased student workload, uncertainty about the semester completion, and confusion about study expectations, which resulted in higher stress levels (Stathopoulou et al., 2020; Van de Velde et al., 2020). Due to the limited social life during the pandemic, these students have also reported feeling lonely, anxious, and depressed (Essadek and Rabeyron, 2020). Prior studies highlighted some coping solutions; for example, students searching for information about the pandemic (Capone et al., 2020; Wathelet et al., 2020) and for meaning in life (Arslan et al., 2020) have higher levels of mental well-being. Students who spend much time on social media platforms and have strong motivation for online learning also report lower levels of distress (Al-Tammemi et al., 2020). Surprisingly, Capone et al. (2020) found no significant deviation in levels of stress and mental well-being from the accepted norm among college students in Italy.

These and other researchers (e.g., Li et al., 2020; Zhai and Du, 2020) call for better understanding of the impact of COVID-19 on student psychological states. First, colleges and universities across the globe need to identify and adopt strategies and resources to address the impact of COVID-19, which is likely to be long lasting. These strategies would include a revision of the existing practices and interventions at the curricular, co-curricular, and extracurricular levels (e.g., Yamada and Victor, 2012; Maybury, 2013; Kareem and Bing, 2014; Mokgele and Rothman, 2014) and at the university-wide level (Mahatmya et al., 2018). Second, COVID-19 has created much uncertainty about “a new normal” in student learning and university functioning. Currently, when most countries are still responding to the pandemic, it seems possible, if not likely, that the change to online or hybrid modes of learning will become more prevalent in colleges and universities across the globe. Therefore, new strategies and resources need to be developed to improve student well-being in the online or hybrid environment. Third, to find effective strategies and resources, colleges, and universities have to identify and understand factors and mechanisms through, which COVID-19 affects student well-being. Consequently, this study sought to examine how student perceptions of their degree completion and future job prospects during the pandemic impact their well-being and what role university support plays in this relationship. To achieve this goal, the study used four scales to collect self-reported data from students in four countries, such as France, Germany, Russia, and the United Kingdom (UK).

Our research contributions are three-fold. First, the study contributes to the emergent knowledgebase of the impact of COVID-19 on student well-being in general (e.g., Al-Tammemi et al., 2020; Capone et al., 2020; Li et al., 2020) and student well-being in France, Germany, Russia and UK in particular (e.g., Essadek and Rabeyron, 2020; Kohls et al., 2020; Savage et al., 2020). Our findings could contribute to the research on the impact of COVID-19 on students and help the higher education sector internationally develop appropriate strategies. Second, this study identifies the key factors affecting students and their learning during the lockdown period and helps understand adjustments needed for the “new normal” learning environment. We argue that the change to an online or hybrid mode of learning will be the “new normal” for teaching, and, hence, we need to explore and find evidence for students to effectively deal with and learn in an online and hybrid environment. Third, using the conservation of resources theory (CoR; Hobfoll, 1988, 1989), we enrich the application of prior student well-being research and provide a theoretical framework that helps understand the mechanism of university support on student well-being.

In the following sections, we introduce the concept of student well-being, provide an overview of the CoR theory (Hobfoll, 1988, 1989), and review resources that universities provide to enhance student well-being. Then we develop hypotheses, describe the study methodology, and present the results and discussion. We conclude with research limitations and future research direction.

## Theoretical Background and Hypotheses Development

### Conservation of Resources Theory

The CoR theory (Hobfoll, 1988, 1989) suggests that people experience stress when they feel the threat of resource loss, a real net loss of resources, and/or a lack of gained resources after resource investment. Two types of resources are examined by this theory. On the one hand, individuals' external resources are object resources (e.g., for university student, laptop for taking online courses, living expenses), social resources (e.g., family help), and condition resources (e.g., stable internet and digital support offered by the university). On the other hand, individuals' internal resource includes personal resources (e.g., self-efficacy and self-control during distance learning) and energy resources (e.g., time and health; Chen et al., 2015; Hagger, 2015). The CoR theory is relevant to better understand the impacts of Covid-19 on university students' well-being as they need to follow fully or partially online courses, they are forced to reduce the social activities to the minimum level, and they should try to manage daily life in the new normal. Simultaneously, Covid-19 remains an international threat to both life and economies, resulting in widespread public nervousness This continuing global pandemic concurrent with the changes in university life are likely to decrease student well-being.

Applying the CoR theory to the current pandemic, Ojo et al. (2020) found that individual reaction and subsequent response to the crisis varies. Some people can bounce back easily and shortly (Luthans et al., 2006; Malik and Garg, 2020) while some people will develop the symptoms such as depression or other psychiatric disorders. University students who are able to optimize the resource gains, cope with changes in daily life, and manage their emotions are more likely to perceive the crisis positively. This in turn not only shows their current level of resilience but additionally enables them to develop their resilience capability. Within this dynamic process, their resilience has served to reduce the stress (Vinkers et al., 2020). In this vein, while students are balancing the resource gains (e.g., university support) and resource loss (e.g., change-related stressors), they show different levels of resilience and which affect their capability to maintain well-being.

### Student Well-Being

Some researchers explain well-being in terms of equilibrium by stating that everybody has a baseline of happiness. According to Headey and Wearing (1991), resources, psychic incomes, and subjective well-being are in a dynamic equilibrium. This equilibrium comprises “physical well-being, plenty of physical resources; absence of fatigue; psychological well-being and evenness of temper; freedom of movement and effectiveness in action; good relations with other people” (Herzlich, 1974, p. 60). From this perspective, well-being could be defined as the balance point between an individual's resource pool and the challenges faced (Dodge et al., 2012; Chen et al., 2015).

During their program completion under the impacts of COVID-19, students face numerous challenges, demands, and turbulences that influence their well-being. For example, they experience diverse social and economic pressures (Wood et al., 2018), have to balance their education, family, and work responsibilities (Moate et al., 2019), and encounter social isolation, discrimination, language barriers, and cross-cultural differences (Daddow et al., 2019). To successfully address these demands and succeed in their pursuit of education and a profession, students at all levels of education and across all disciplines have to have timely and adequate resources (Mokgele and Rothman, 2014; Wood et al., 2018). These resources help to address students' needs and, hence, reduce their burn-out and stress and increase their engagement in learning activities, meaning making, and life satisfaction (Flinchbaugh et al., 2012).

Universities can deploy these resources via curricular, co-curricular, and extracurricular activities (Flinchbaugh et al., 2012; Yamada and Victor, 2012; Maybury, 2013). In the classroom, clear assessment criteria, classroom policies, and project deadlines can eliminate student frustration, dissatisfaction, and withdrawal from active learning (Mokgele and Rothman, 2014). Sports and physical activity have also been shown to decrease depression and stress and increase student well-being (Yazici et al., 2016). Campus libraries contribute to promoting student well-being by ensuring easy access to learning resources and a learning space for all students (Cox and Brewster, 2020). These practices can also help students to increase intrinsic motivation to learn, voice their concerns, enact their identities, and make sense of their experiences. In contrast, a campus environment that does not efficiently address unhealthy and unethical social interactions, for example, bullying (Chen and Huang, 2015), cyberbullying (Musharraf and Anis-ul-Haque, 2018), and cyber dating abuse (Viillora et al., 2020) increases student depression and anxiety and decreases student quality of life. This can lead to students starting to feel less happy and less intrinsically motivated to learn, which affects their well-being.

### The Perceived Impact of COVID-19 on Degree Completion and Student Well-Being

During COVID-19, more than 100 countries implemented either nationwide or local “lock-down” measures at least once. Such closures meant that face-to-face courses have been transitioned to online learning (Kwok et al., 2020). The impact of COVID-19 on student life becomes significant. These can be, for example, experiencing more workload, adapting oneself to an online learning mode immediately, or moving back to home without sufficient preparation but can also include more worries due to uncertainty and fear of pandemic. In addition, the impact of COVID-19 on each student varies. Some students have limited access to connectivity; some do not have adequate IT equipment to attend online classes, and others cannot afford the extra cost to improve their IT resources (UNESCO, 2020). Meanwhile, students' subjective socioeconomic loss affects their life outcomes. In their study, Kohls et al. (2020) argue that income changes during the pandemic affect the levels of depressive symptoms. In other words, socioeconomic loss leads to increasing stress. For instance, many students rely on part-time jobs to gain their living expenses, and due to the lockdown and economic crisis, they either cannot get a renewed contract or they become unemployed. Unemployment leads not only to earning loss, but also to psychosocial asset loss, social withdrawal, and psychological and physical well-being loss (Brand, 2015). All in all, the unavailable external resources can impact the student learning experience, for example, interrupted learning, lack of participation in in-class discussion, absenteeism in class, and restraints to taking their final exams, all of which can result in students accepting lower-status jobs in order to survive. Additionally, some students have also faced discrimination (Hardinges, 2020) during COVID-19, which may lead to mental health problems (Kang et al., 2020). Students from minority groups (e.g., Asian students, in particular the Chinese) have encountered social isolation and stereotypes, which could impact their student experience and job prospects.

Furthermore, the impact of the COVID-19 outbreak on the world has been substantial. With insufficient knowledge of the virus and no available vaccine for months, students may be prone to develop more negative emotions. Prior studies have shown that negative emotions have a critical impact on well-being (Gross, 2015; Puente-Martínez et al., 2018). Students may experience real and potential loss of resources and a mismatch between task demand at the universities and their resource availability (Hagger, 2015). With the increasing negative emotions, their well-being could be affected as they become more concerned about the impact of COVID-19 on their studies.

We, therefore, predict that COVID-19 would lead to students' negative well-being because students may experience more stress related to uncertainties in their academic success, negative economic impact, and lack of perceived support (Cao et al., 2020). Meanwhile, students would feel the need to deploy more time and energy to protect themselves against and recover from resource loss (Hobfoll et al., 2018) in order to avoid putting their well-being at risk. We propose the following hypothesis:

H1: The perceived impact of COVID-19 on student concerns for degree completion will negatively predict levels of student well-being.

### The Perceived Impact of COVID-19 on Student Concerns for Future Job Prospects and Student Well-Being

COVID-19 has triggered a worldwide economic recession (OECD, 2020). With the lockdown measures implemented by many governments, business opportunities become restricted in many sectors and unemployment is rising. Many companies have reported layoffs. As predicted during the first wave of the pandemic by OECD (2020), the second wave of infections in late 2020 worsened the economic situation, and more companies suffered from the economic crisis, which has impacted job losses, financial well-being, and standards of living. As a result, students search for job opportunities to ensure their return on education investment would be limited. Thus, there are more job demands than supply. According to the CoR theory, when resources are lost or perceived to be threatened, people experience stress and are motivated to gain back their resources (Baer et al., 2018). Under the economic lockdown and recession, more students may have difficulties in finding jobs and/or internships, which could negatively affect students' self-esteem (personal resource) and their individual economic well-being (object resource) for instance. Without a guarantee to job prospects, students feel more stressed about their future and return on education investment, which decreases their engagement in learning activities and increases their negative emotions (Flinchbaugh et al., 2012). Therefore, the more concerned students feel about the impact of COVID-19 on their future job prospects, the lower their level of well-being and the higher the level of negative affect. We suggest the second hypothesis:

H2: The perceived impact of COVID-19 on student concerns for future job prospects will negatively predict levels of student well-being.

### The Mediating Role of University Support

Universities play an important role in ensuring and increasing student well-being. In the classroom, specific interventions, including positive psychology assignments (Maybury, 2013), stress management and journaling (Flinchbaugh et al., 2012), and mindful awareness practices (Yamada and Victor, 2012) have been shown to improve student well-being. A supportive and enabling environment on campus has been proved to ensure student well-being (Kareem and Bing, 2014; Daddow et al., 2019) by fostering their sense of belonging, positive relationships with others, autonomy, and competencies (Baik et al., 2016). For example, through informal social interactions students explore and relate to individual, group, and even the entire university values, which increases their well-being (Sortheix and Lönnqvist, 2015). Mahatmya et al. (2018) describe a set of integrated and interrelated courses that incorporate both traditional and experiential learning activities for undergraduate students. To monitor and manage student well-being outside the classroom, universities provide other services and interventions, including, for example, stress management (Mokgele and Rothman, 2014), counseling (Kareem and Bing, 2014), inter-faith, and cultural diversity programs (Daddow et al., 2019). In summary, these services and interventions represent the support that students can access and, therefore, can make students feel more positive about their resource gains. The perceived impact of COVID-19 may result in students perceiving university support to be limited, insufficient, or non-existent. Therefore, students would need extra resources to achieve the university success and increase their well-being. Therefore, we propose the following hypothesis:

H3a: University support will mediate the relationship between the perceived impact of COVID-19 on student concerns for degree completion and levels of student well-being.

Similarly, students need support from their universities to increase their chances of employment before and upon graduation (McMurray et al., 2016; Donald et al., 2018). These are activities and initiatives provided by academic and student services, campus libraries and student organizations to help students cope with the study demands, develop professional networks, practice job interview skills, write resumes, and gain internships. However, COVID-19 has greatly impacted these resource offering. For example, career services would typically provide more support in a face-to-face format (e.g., career fairs and case championships), but now universities may face difficulties (e.g., time, money, and available talent) to develop effective comparable online services. If universities help students find jobs and internships, students could feel supported, less stressed, and more optimistic about their future careers. Hence, we propose the following hypothesis:

H3b: University support will mediate the relationship between the perceived impact of COVID-19 on student concerns for future job prospects and levels of student well-being.

## Methodology

### Sample and Procedure

The sample was collected from university students in France, Germany, Russia, and UK between April and June, 2020. In total, 2,707 questionnaires were collected. However, 765 had missing values; after removing them, 1,932 observations were included for further analysis. Out of these 1,932 participants, 119 were recruited from UK, 227 from Russia, 1,314 from Germany, and 272 from France (see Table 1). From the students in the sample 63.8% were female, 35.8% male, and 0.4% other. The mean age was 22.87 years old. Most students lived at home (68.5%) and studied full-time (85.1%). Over half of the respondents were first- and second-year undergraduate students.

**Table 1:** Demographics.

**Variables**		**Frequency**	**Percent**
Country	Russia	227	11.7
	The UK	119	6.2
	Germany	1,314	68.0
	France	272	14.1
	Total	1,932	100.0
Gender	Male	691	35.8
	Female	1,233	63.8
	Other	8	0.4
Residing	Home	1,324	68.5
	Still on campus	81	4.2
	With friends	36	1.9
	With family	432	22.4
	Other	59	3.1
Study mode	Full time	1,644	85.1
	Part time	204	10.6
	Other	84	4.3
Study year	Undergraduate year 1	559	28.9
	Undergraduate year 2	519	26.9
	Undergraduate year 3 (Placement/Study Abroad)	148	7.7
	Undergraduate year 3 (Final year)	277	14.3
	Undergraduate year 4 (Final year)	122	6.3
	Post-graduate year 1	192	9.9
	Post-graduate year 2	96	5.0
	Post-graduate other	19	1.0

The questionnaire was administered with Qualtrics XM software. Participants received the link and filled in the questionnaire individually, voluntarily, and anonymously. The project followed ethical standards of research required by each participating university.

### Measures

The first part of the self-reported questionnaire consisted of demographic details such as gender, age, country, place of residence, study mode, and study year. The main part of the questionnaire included the following four scales.

#### University Support

University support was measured by asking students to rate to which extent they got support from their lecturers and universities. Two items reflected university support and were measured on a 5-point Likert scale (e.g., Please rate these as they apply to your current experience: I get support that I need from the following:—My lecturers). This was based on the social support scale developed by Pierce et al. (1991). Good internal consistency was achieved (α = 0.72).

#### Well-Being

Well-being can be conceptualized as having such components as valence and intensity (Warr, 2003). Therefore, two scales were used to capture well-being in different states: in the moment and general.

##### In the Moment Well-Being

To test the valence of student well-being in response to predictors, it is important to represent well-being in terms of independent dimensions of positive and negative emotional states (Tellegen et al., 1999). In the moment well-being was measured by a 5-point Likert scale developed and validated by Russell and Daniels (2018). This scale helps to measure specific positive and negative emotional states relevant to a particular event in time, or “right now.” This ensures affect is measured at its lowest level in terms of duration demonstrating a specific emotional response (Frijda, 1993). Examples of positive states include happy, motivated, and active; examples of negative states include anxious, annoyed, and tired. Good internal consistency was found for negative (α = 0.70) and positive (α = 0.79) dimensions.

##### General Positive Well-Being

To draw comprehensive conclusions as to the effects of predictors on student well-being, it is necessary to also use a summative circumplex model of well-being (Feldman Barrett and Russell, 1998). This measures the second level of mood-based affect that is not directly anchored to an event and, therefore, at a different intensity to momentary affect (Brief and Weiss, 2002). General positive well-being was measured with World Health Organization (1998) 5-point Likert scale ranging from “strongly disagree” to “strongly agree.” This scale helps to assess student mood-based affect for the past 2 weeks. A sample item is “I have felt cheerful and in good spirits and I have felt calm and relaxed.” Good internal consistency was found (α = 0.84).

#### Student Concerns

This scale was devised to assess participants' concerns about the impact of COVID-19 on the basis of seven items. The items were rated on a 5-point Likert scale ranging from “not at all stressed” to “extremely concerned.” Varimax orthogonal rotation with Kaiser normalization was used for factor analysis extraction. All factors with eigenvalue >1, explaining 60% of the variance, were considered for further analysis. Coefficients smaller than 0.5 were excluded to get a reasonable number of factors with larger share of variance (Field, 2009). Adequacy of sample size measured by KMO and Bartlett's test of sphericity established a test score of 0.818 (*p* < 0.001). Communalities for variables taken for analysis were >0.5. Based on the dimension reduction technique, two latent variables were found to account for 77.38%, so the following two subscales were identified:

*Concerns for degree completion* measured the perceived effect of COVID-19 on student ability to complete their degree and meet academic expectation. The following four items comprised the subscale: “my exams and assessments,” “my ability to complete my course,” “my final degree/course qualification grade,” and “my grades.” This subscale had a good internal consistency (α = 0.89).

*Concerns for future job prospects* measured the perceived effect of COVID-19 on student ability to become employed upon graduation. These three items comprised the subscale: “my employability,” “the wider economy,” and “job prospects.” This subscale had a good internal consistency (α = 0.86).

### Data Analysis

The Statistical Package for Social Sciences software version (26) with AMOS was used to analyze the data. Descriptive analysis was used to determine means, standard deviations, confidence intervals, skewness, and correlations among the six main variables (see Table 2).

**Table 2:** Descriptive statistics, correlations, and reliability coefficients.

**Variables**		***M***	***SD***	**95% confidence interval for mean**		**Skewness**	**Cronbach's α coefficients**	**1**	**2**	**3**	**4**	**5**
				**Lower**	**Upper**							
1.	University support	3.64	1.01	3.60	3.69	−0.61	0.72					
2.	General WB	2.83	0.95	2.79	2.87	0.19	0.84	0.340^**^				
3.	Positive in the moment WB	2.76	0.74	2.73	2.80	0.42	0.79	0.313^**^	0.698^**^			
4.	Negative in the moment WB	2.70	0.75	2.67	2.74	0.35	0.70	−0.268^**^	−0.565^**^	−0.513^**^		
5.	Concerns for degree completion	3.18	1.15	3.13	3.23	−0.17	0.89	−0.346^**^	−0.504^**^	−0.463^**^	0.473^**^	
6.	Concerns for future job prospects	3.08	1.24	3.02	3.13	−0.04	0.86	−0.057^*^	−0.260^**^	−0.208^**^	0.293^**^	0.407^**^

*M, mean; SD, standard deviation; N = 1,932; WB, well-being*;

* *p < 0.05, two-tailed*;

** *p < 0.01, two-tailed. The numbers in the title row correspond to the numbers of variables in the first column*.

Since the purpose of this study was to understand the antecedents of well-being, a path analysis was performed by employing structural equation modeling with maximum likelihood estimation method. The use of structural equation modeling in social science and education when testing mediation is recommended as it allows to test multiple pathways to assess the viability of the hypothesized model (Wu and Zumbo, 2007).

The study was exploratory; therefore, two types of university concerns served as independent variables: support from university as a mediating variable and general well-being together with either negative or positive in the moment well-being as the dependent variables. To determine model fit, we applied two types of fit indices: absolute fit measures (χ^2^, RMSEA, AGFI) and incremental fit measures [NFI, NNFI (TLI), CFI; Hooper et al., 2008]. Chi-square (χ^2^) in the range between 2.0 and 5.0 and the probability level with insignificant *p*-value (*p* > 0.05) were acceptable for threshold levels. The root mean square error of approximation (RMSEA) in the range of 0.03–0.08 provides a good fit. Values >0.95 were suitable for the adjusted goodness-of-fit statistic (AGFI), normed-fit index (NFI), Tucker-Lewis index in AMOS (TLI) or non-normed fit index in EQS (NNFI), and comparative fit index (CFI; Hooper et al., 2008).

## Results

**Figure 1:**
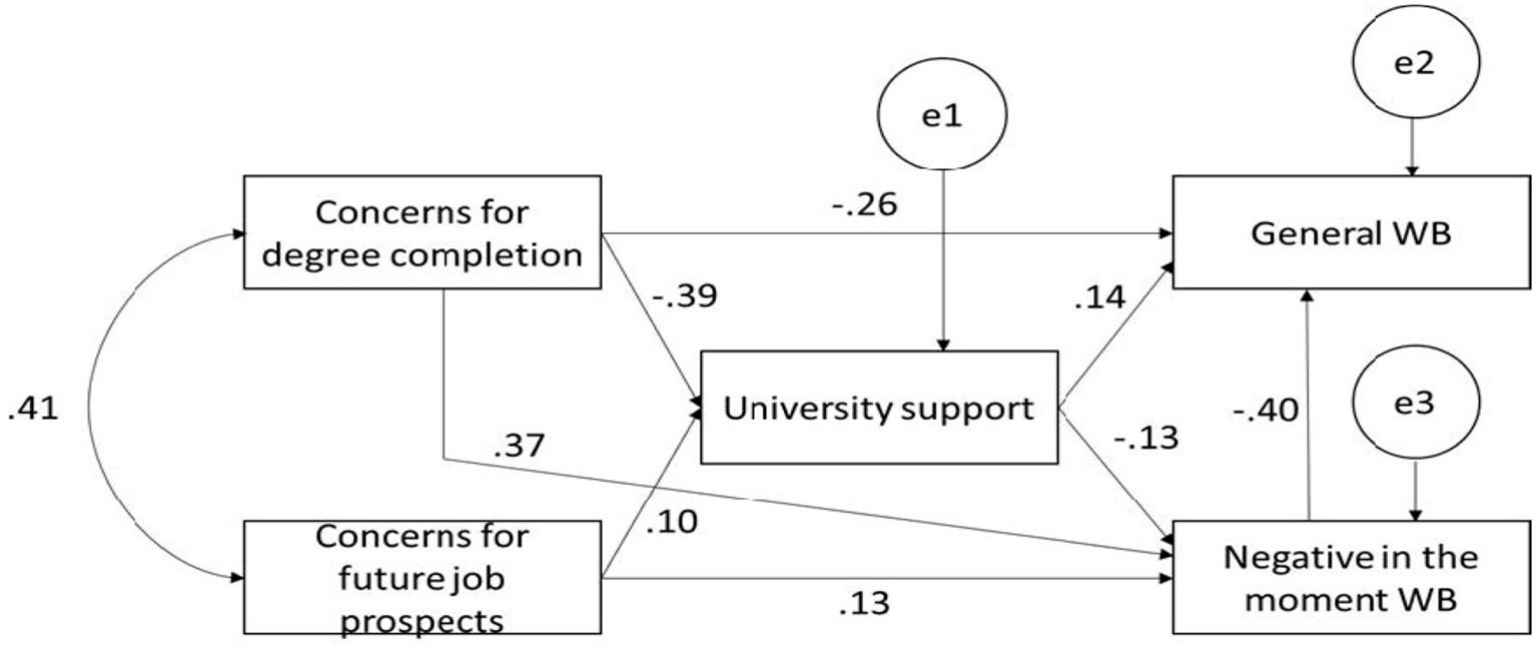
Path analysis with negative in the moment well-being.

First, path analysis was run to further evaluate the relationships between student concerns for degree completion and future job prospects, university support, general well-being, and negative in the moment well-being. Path analysis was also used to test the mediation model in terms of overall fit. The model shows satisfying results with the following model fit statistics: *p* = 0.089, χ^2^ = 2.901, RMSEA = 0.031, AGFI = 0.991, NFI = 0.999, NNFI (TLI) = 0.991, CFI = 0.999, and path coefficients presented in Figure 1.

**Figure 2:**
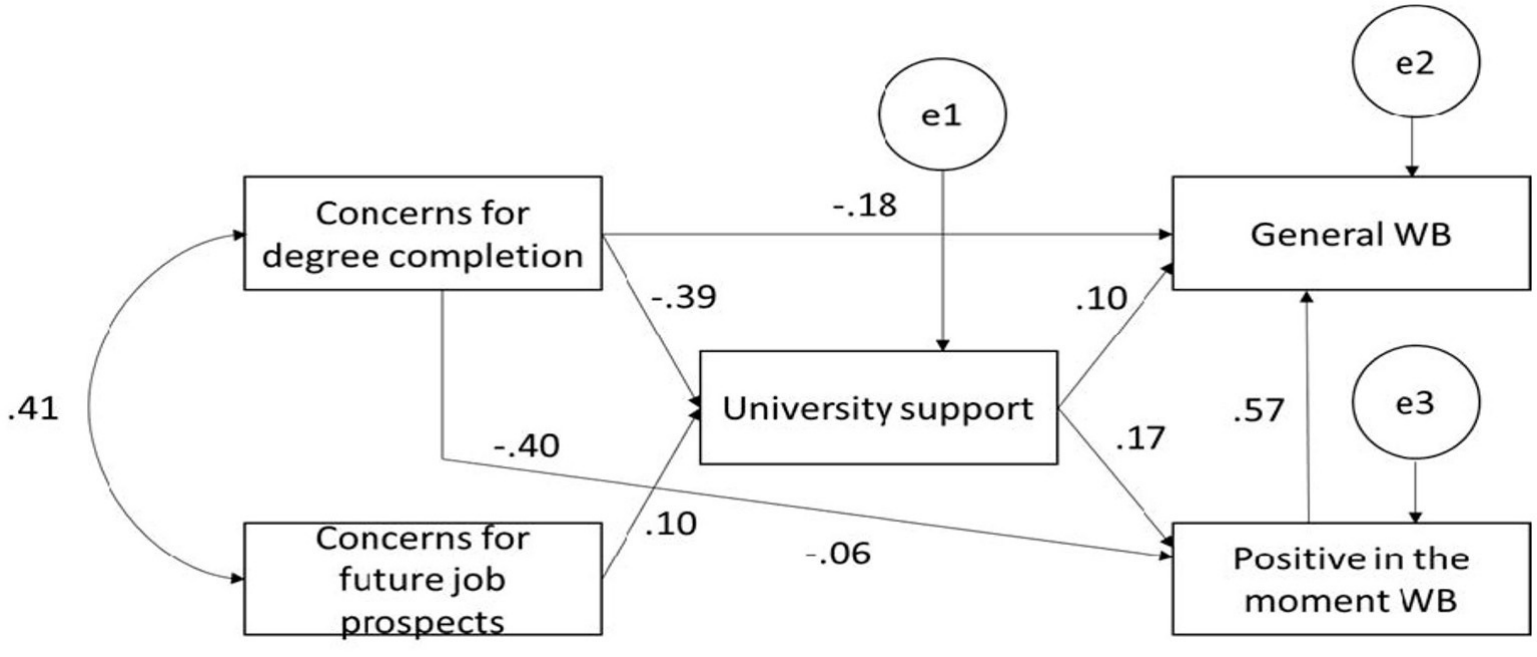
Path analysis with positive in the moment well-being.

Second, similar analysis was performed to explore the relationships between student concerns, university support, general well-being, and positive in the moment well-being. This model demonstrates the following statistics: *p* = 0.055, χ^2^ = 3.677, RMSEA = 0.037, AGFI = 0.989, NFI = 0.999, NNFI (TLI) = 0.990, CFI = 0.999 (see Figure 2).

All coefficients were significant beyond 0.05 level. The analyses of direct, indirect and total effects of student concerns on general well-being and both negative and positive in the moment well-being are shown in Tables 3, 4, respectively.

**Table 3:** Direct, indirect, and total effects of student concerns on general well-being.

**Paths**	**Coefficients for path analysis with negative in the moment WB**	**Coefficients for path analysis with positive in the moment WB**
CDC → General WB	−0.264	−0.181
CDC → University support → General WB	−0.225	−0.306
Total	−0.490	−0.487
CFJP → General WB	–	−0.062
CFJP → University support → General WB	−0.034	0.02
Total	−0.034	−0.043

*CDC, student concerns for degree completion; CFJP, student concerns for future job prospects; WB, well-being*.

**Table 4:** Direct, indirect, and total effects of student concerns on in the moment well-being.

**Paths**	**Coefficients for path analysis with negative in the moment WB**	**Coefficients for path analysis with positive in the moment WB**
CDC → in the moment WB	0.374	−0.403
CDC → University support → in the moment WB	0.051	−0.067
Total	0.424	−0.47
CFJP → in the moment WB	0.133	
CFJP → University support → in the moment WB	−0.013	0.017
Total	0.12	0.017

*CDC, student concerns for degree completion; CFJP, student concerns for future job prospects; WB, well-being*.

### The Perceived Impact of COVID-19 on Student Concerns for Degree Completion and Student Well-Being

The direct effect of student concerns for degree completion on general well-being and positive in the moment well-being is significant and negative (−0.18 and −0.40, respectively). However, when we consider negative in the moment well-being, concerns for degree completion had negative direct effect on general well-being (−0.26) and positive in the moment well-being (0.37). Moreover, the analysis of indirect effects demonstrates that university support mediates the effect of concerns for degree completion on general well-being (−0.31) and positive in the moment well-being (−0.07). In the same way, this construct influences negative in the moment well-being affect (0.05) and general well-being (−0.23). These results suggest that the perceived impact of COVID-19 on concerns for degree completion has a significant negative effect on student well-being while university support plays a mediating role between these two variables, therefore fully supporting H1and H3a.

### The Perceived Impact of COVID-19 on Future Job Prospects and Student Well-Being

Concerns about the impact of COVID-19 on future job prospects have a direct effect on general well-being, which is significant and negative (−0.06), together with positive in the moment well-being and a significant positive effect on negative in the moment well-being (0.133). These results suggest that increased levels of concerns about the effect of COVID-19 on future job prospects leads to lower levels of general well-being and higher levels of negative in the moment well-being. Therefore, H2 is partially supported. Furthermore, university support attenuates the effect of concerns about future job prospects on negative in the moment well-being (−0.013) (Table 4). These results support H3b, thereby suggesting university support has a beneficial effect on student well-being.

Regarding the future job prospects, degree completion, and well-being, we ran the analysis of variation (ANOVA) to understand the differences between undergraduates (*n* = 1,625) and post-graduates (*n* = 288) separately. Post-graduates did not show any significant differences regarding degree completion [*F*_(1, 286)_ = 0.065, *p* = 0.798], future job prospects [*F*_(1, 286)_ = 0.585, *p* = 0.445], and general well-being [*F*_(1, 286)_ = 0.626, *p* = 0.430]. However, significant differences between the undergraduate groups were observed for all three variables, namely, concerns for degree completion [*F*_(4, 1, 620)_ = 7.77, *p* < 0.001], future job prospects [*F*_(4, 1, 620)_ = 30.2, *p* < 0.001], and general well-being [*F*_(4, 1, 620_) = 4.99, *p* < 0.001]. Then, a year-by-year comparison analysis was performed by applying Tukey's honestly significant difference test to examine how this is impacted by the year of study. As a result, first-year undergraduates (3.34 ± 1.09 min) expressed significantly higher levels of concerns for degree completion than third- (2.99 ± 1.17 min, *p* < 0.001) and fourth-year (2.98 ± 1.29 min, *p* = 0.01) students. Similarly, second-year undergraduates (3.34 ± 1.12 min) expressed significantly higher levels of concerns for degree completion than third- (2.99 ± 1.17 min, *p* < 0.001) and fourth-year (2.98 ± 1.29 min, *p* = 0.01) students. However, the findings were opposite when we compared the future job prospects means between years of study. The fourth-year students (3.76 ± 1.18 min) demonstrated higher significant concerns in comparison with other undergraduate groups, namely first-year (2.71 ± 1.12 min, *p* < 0.001), second-year (2.89 ± 1.25 min, *p* < 0.001), and even third-year (3.33 ± 1.26 min, *p* = 0.007) as well as those who study abroad or through placement programs (3.31 ± 1.15 min, *p* = 0.016). As for general well-being, the most optimistic group was undergraduates who participated in placement programs or studied abroad. These respondents expressed significantly higher levels regarding general well-being over the past week (3.09 ± 0.93 min) than first-year (2.84 ± 0.92 min, *p* = 0.039) and second-year (2.72 ± 0.97 min, *p* < 0.001) students. However, there were no statistically significant differences between placement/study abroad undergraduates and third-year (2.85 ± 0.94 min, *p* = 0.095) and fourth-year (2.93 ± 0.92 min, *p* = 0.641) students.

## Discussion

The purpose of this study was to examine how student perceptions of their degree completion and future job prospects during the pandemic impact their well-being and what role university support plays in this relationship. We developed and tested the relationship between the perceived impact of COVID-19, university support, and student well-being. Our results showed that the perceived impact of COVID-19 on student concerns for degree completion negatively predicts levels of student well-being. In other words, the more worried students are about the impact of COVID-19 on their studies, the more their levels of well-being decrease. This result is in line with the findings of Poots and Cassidy (2020) who found support to be a positive predictor of well-being and a significantly negative relationship between academic stress and support. COVID-19 disrupted the balance point between the students' resource pool relevant to their academic pursuits and the numerous challenges they face (Dodge et al., 2012). Programs, processes, and services have gone online leading to student poor well-being. Therefore, the impact of the pandemic, and similar crises, extends beyond student perceptions of their success in their main role as students but also to their perceptions of happiness (Pollard and Lee, 2003), life satisfaction (Diener and Diener, 1996), and being intensely alive and authentic (Ryan and Deci, 2001).

Also, the results revealed that the relationship between the perceived impact of COVID-19 on student concerns for degree completion and levels of student well-being is mediated by university support. This result illustrates the importance of university support on student perceptions and emotional states, including stress, meaning making, and life satisfaction (Flinchbaugh et al., 2012). This university support represents a resource that is outside of individuals (Hobfoll et al., 2018). When this support is timely and adequate (Mokgele and Rothman, 2014; Wood et al., 2018), students can successfully deal with the demands of their educational pursuits. However, the study also indicates that when students perceive the negative impact of COVID-19 on their degree completion and well-being, they are less likely to perceive their university as supportive. We explain this situation with the different perceptions in effective support. Students and universities have differences in their views about which priorities support well-being (Graham et al., 2016). Students perceive university support as valuable and effective when they can obtain lecturers' timely feedback to their emails, transparent, and fast communication in relation to the changes from the COVID-19 situation, dynamic online courses, and emergency financial support amongst other factors. Students are becoming more exigent on the resources that universities could offer to support their academic success and how efficiently the support is delivered. From the university perspective, they need to develop solutions that are in line with institutional or governmental measures, but little concrete information exists. Universities may find it difficult to cope with changes related to COVID-19 immediately (e.g., adopt fully online learning environments whilst not all the lecturers have the capabilities or facilities to teach online). Therefore, students perceive that university support is not sufficient to their academic success while universities have already made great efforts to ensure online learning and working-from-home policies. Given that students' immediate priority is their academic performance, they are trying to gain more educational resources than universities may be able to offer. Students, therefore, may perceive their university support as insufficient to their degree completion. This could also be explained by one of the principles of the CoR theory that states that resource loss is disproportionately more prominent than resource gain (Hobfoll et al., 2018). Therefore, students seem to be very sensitive to a lack of or very little immediate and long-term university support to their academic success.

The study also found unexpected results related to the student perceptions of their future job prospects. First, there is no direct relationship between the perceived impact of COVID-19 on future job prospects and student well-being. In other words, student concerns about the impact of COVID-19 on their future job prospects does not decrease their level of well-being. This result needs further research. It is possible to suggest that students do not see an immediate threat because job prospects are about the future (Xu et al., 2015). For instance, students that are not in their final academic year could feel less of a threat of resource loss in terms of future employment. Instead, they are more stressed and concerned about the impact of the pandemic on their degree completion that is more urgent at the moment. Interestingly, students who are more stressed about the impact of COVID-19 on their future job prospects are more likely to perceive their university as giving higher levels of support. As fewer employment opportunities exist in the labor market, students expect university networks to offer them some potential job opportunities.

The study also showed that students at different levels of education perceived the impact of the pandemic in different ways. The most vulnerable group was undergraduates who expressed significantly higher levels of concerns for degree completion. Perhaps, due to the uncertainty related to the duration of lockdowns, social distance measures, and other restrictions as well as vaccine effectiveness and availability, first year students struggled to see how they are able to complete their program the most. They also have fewer life experiences to cope with different types of stress that appeared simultaneously. At the same time, last year students struggled the most with potential job prospects. This is somewhat expected because this group of students usually tries to find full-time jobs upon the degree completion. University management can mitigate these student concerns by introducing relevant practices based on the student study year.

### Theoretical Implications

This study offers several contributions to better understand the mechanism of university support on student well-being during the COVID-19. First, our findings are in line with the prior studies on the relationships between stress and well-being, and support and well-being. The research on the impact of COVID-19 on student concerns for degree completion and job prospects is underdeveloped. Therefore, by examining student resource loss, we have extended the application scope of the CoR theory and enriched COVID-19 related research.

Second, our findings highlight that students may not perceive university support in the same way when it is related to their concerns for degree completion or job prospects. Prior studies have acknowledged the positive relationship between university support and student well-being (Baik et al., 2019). Our findings imply that perceived effective support is context-specific. Under the impact of COVID-19, all students are concerned about their academic performance and are more exigent on university support. When students feel that they are not able to get support to achieve the balance between resource investment (e.g., spending more time to work online for group-based activities) and the challenge of continuing with their studies (e.g., receiving no immediate feedback when they have inquiries for lecturers or administrators), they may have a lower level of well-being (Dodge et al., 2012). To mitigate the risk to their well-being, students feel the need to deploy more time and energy to protect themselves against resource loss and recovery (Hobfoll et al., 2018).

Third, this study assessed negative in the moment well-being. Our results show that university support could mediate the relationship between impacts of COVID-19 (both on degree completion and job prospects) and student well-being. However, when students perceive a high level of support from the university, they feel a higher level of well-being and a lower level of negative in the moment well-being. This once again implies that university support plays an important mediating role in student perceptions of well-being.

### Practical Implications

This study confirms the mediating role of university support that helps turning negative impact of COVID-19 into positive feelings of well-being. Universities could increase student well-being by giving support to student studies and their career and job prospects. This support should come from a wide range of university services that are responsible for all aspects of the student learning experience. For example, program faculty and directors should provide students sufficient and timely information about upcoming mandatory internships. Career centers should utilize their partnerships and networks in the local community to assist in finding their first job after graduation and/or internships. This support should include course instructors, program directors, university management and administration, digital and IT support, and supports from partnership universities for international exchange programs. Supervisors and administration should work closely with students conducting research projects related to their theses or dissertations. They should support them in setting the dissertation topic and research questions, data collection and data analysis, discussion of initiation findings, text drafting, and defending.

The study also suggests that a lack of questioning or concerns related to university support from students does not imply that students feel that they are receiving this support. This could indicate that students may feel forgotten, abandoned, or hopeless about receiving support from the university. Therefore, universities should ensure visibility and accessibility of support, which in the context of online learning would require integration and collaboration between academic and university support services (e.g., IT support, career centers, academic advising, and international exchange programs). They help students navigate the support systems and access all the resources they require to succeed academically and professionally. Universities should not only provide the resources needed for students to engage with online learning, but also propose training on different online pedagogies to course instructors, as these two points could ensure more a positive learning experience for students and their well-being outcome. In addition, universities should monitor the student well-being experience and provide relevant resources and interventions.

Also, with online learning, face-to-face social interactions are missing. Therefore, lecturers and administrative staff should concentrate more on relationship building. They should facilitate the online learning experience, adopt clear communication strategies, improve the learning tools (e.g., PowerPoint and recorded lectures) and diversify assessment methods (e.g., moving from traditional exams to video-based oral presentation and using applications to motivate students to engage in online discussions).

From the student perspective, universities should be aware of the students' changing emotional responses from positive to negative during the COVID-19 pandemic. Given that the impact of COVID-19 would probably induce more negative emotional states, universities should offer more support for emotional management. This should encourage students to talk about their concerns, worries, and anxiety toward COVID-19 and to help them destigmatize the fear of COVID-19 on their studies and future. This support should not be a one-time-event, but ongoing. With positive emotions, students are more capable to counterbalance the perceived negative impact of COVID-19 on their degree completion and job prospects by effectively using different resources to reduce resource loss.

Finally, it is important to note that staff well-being is essential in order to support this student learning experience. Therefore, whilst universities propose different support activities to promote student learning, academic performance, and future job opportunities, they should also put in place a variety of resources to support staff. Pedagogy training, digital support, online well-ness programs, high quality information related to Covid-19, peer learning, appreciation attitude, and positive thinking should be promoted. University support and well-being feeling of their staff are a must for their adjustment to this “new normal” work context and a better service to students. It should be acknowledged that although many of the recommendations in this section are best practice in non-crisis times, this research has shown that the current acute pandemic situation and its effect on students (and staff) requires a sustained and reliable response, which utilizes existing policies and procedures to their maximum potential.

### Limitations and Future Research

The study used a cross-sectional design, so the results cannot illustrate the process and evolution of how the identified variables influence student well-being. Considering the nature of the COVID-19 crisis, it would be very useful to develop a longitudinal study. Given the subjective nature of perceptions of well-being, there is an opportunity to extend the research and give a deeper understanding of the students' experience by taking a qualitative study approach. For example, phenomenology could help researchers understand lived experiences of students (van Manen, 1990) during COVID-19. Phenomenology could also help to find out how students experience their well-being or how they “perceive it, describe it, feel about it, judge it, remember it, make sense of it and talk about it with others” (Patton, 2002, p. 104). Further studies could also explore potential variables that may be more likely to show differences in a cross-cultural context, for example, how various types of social support may be perceived differently in various cultural contexts. The study used self-reported data that could have created a certain bias, so future studies should consider using observations and document analysis to triangulate data.

The study found that there were no student concerns about the impact of COVID-19 on their future job prospects and this did not decrease their level of well-being. This result needs further research. For example, there may be some benefits of using a qualitative and cross-cultural approach such as diary methods. A longitudinal study could help tracking how student concerns for their future job prospects change. Many countries have overcome the second wave of COVID-19, but uncertainty about the economy and high unemployment rates remains. Similarly, it would be useful to understand how students address their concerns for their job prospects and employment and search for and obtain jobs.

## Conclusion

The study showed the usefulness of the CoR theory in helping universities and students to understand the emotional responses and impacts on student well-being of the sudden and dramatic changes to the learning experience of an unexpected global crisis. It was found that a major crisis negatively impacts student well-being and their concerns about their studies. However, the longer-term concerns about job prospects and careers had no negative impact on well-being. Support was shown to be an important mediator in the overall impact on student well-being.

## Data Availability Statement

The raw data supporting the conclusions of this article will be made available by the authors, without undue reservation.

## Ethics Statement

The studies involving human participants were reviewed and approved by University of Hertfordshire SSAHEC with Delegated Authority. The patients/participants provided their written informed consent to participate in this study.

## Author Contributions

GP and KM were substantially involved in planning and conducting the study. NV, SN, and SR-T carried out the data analysis. MP, CJ, and DY wrote the article with contributions by NV, GP, SN, and KM. All authors revised the manuscript critically for important intellectual content, read, and approved the submitted version. All authors were involved in distribution of the survey.

## Conflict of Interest

The authors declare that the research was conducted in the absence of any commercial or financial relationships that could be construed as a potential conflict of interest.
